# Relation of Moderate Physical Activity to Blood Markers of Oxidative Stress and Antioxidant Defense in the Elderly

**DOI:** 10.1155/2019/5123628

**Published:** 2019-02-11

**Authors:** Mariusz Kozakiewicz, Rafał Rowiński, Maciej Kornatowski, Andrzej Dąbrowski, Kornelia Kędziora-Kornatowska, Aneta Strachecka

**Affiliations:** ^1^Department of Food Chemistry, Nicolaus Copernicus University Collegium Medicum, Bydgoszcz, Poland; ^2^Faculty of Agrobioengineering, Department of Tourism and Recreation, University of Life Sciences in Lublin, Lublin, Poland; ^3^Department and Clinic of Geriatrics, Nicolaus Copernicus University Collegium Medicum, Bydgoszcz, Poland; ^4^Faculty of Physical Education, Halina Konopacka Higher School of Physical Culture and Tourism in Pruszkow, Poland; ^5^Faculty of Biology, Animal Sciences and Bioeconomy, Institute of Biological Basis of Animal Production, University of Life Sciences in Lublin, Poland

## Abstract

The aim of the present study was to establish whether markers of oxidative stress and the enzymatic defense system of the blood are related to moderate physical activity in younger old and the oldest old men. They were divided into four groups according to the age and level of physical activity (groups YN and YA—inactive and active younger old men aged 65-74 years, groups ON and OA—inactive and active oldest old men aged 90-99 years). Venous blood was collected from the subjects in the morning before breakfast. MDA concentration and antioxidant enzyme activities (SOD, CAT, GPx, and GR) in erythrocyte hemolysates were assayed. The concentration of isoprostanes (8-iso-PGF2*α*) and carbonyl groups in protein (CP) was measured in plasma and serum. All assayed antioxidant enzyme activities and the SOD/GPx ratios were significantly higher in the active younger old males than in all the inactive ones. In the group of oldest old active participants, only the GPx activity was significantly higher compared to the inactive oldest old males. The activity of CAT and GPx in the younger old inactive men was significantly lower than that in the oldest old inactive subjects. However, SOD, CAT, and GR activities and SOD/GPx ratio were significantly higher in the younger old active men compared to the oldest old active participants. The concentrations of isoprostanes, protein carbonyls, and MDA were significantly lower in both active and inactive younger old males than in the respective groups of the oldest old men and in both groups of active men, independently of age, compared to the respective inactive subjects. The present study confirmed that oxidative stress is related to age. Physical activity caused a decrease of oxidative stress markers independently of age and resulted in an increase of GPx activity in both younger old and the oldest old active groups.

## 1. Introduction

It is well known that physical activity has the health benefit, even in the elderly, when many physiological functions are in decline, and it reduces susceptibility to chronic diseases of the cardiovascular and other systems [1, 2]. Recommendations of exercise for older persons differ and depend on individual needs and abilities which should be matched with the type of activity, in terms of intensity, duration, and frequency of exercise [3–6].

According to the free radical theory of ageing, reactive oxygen species (ROS) generated during physiological processes, mainly in mitochondrial electron transport, can oxidize lipids, proteins, and DNA molecules, resulting in oxidative stress, because the antioxidant potential (the enzymatic as well as the nonenzymatic system) is insufficient to prevent these impairments [7, 8]. The greater the ROS generation, the more the concentration of protein carbonyls and products of lipid peroxidation—isoprostanes and malondialdehyde (MDA) increase [7, 8]. On the other hand, it was established that reactive oxygen species play an essential role in cellular homeostasis as signaling molecules and mediate many physiological processes [9–11].

It is well known that oxidative stress is an important factor contributing to the development of chronic inflammation, and there is an association between elevated inflammatory markers and chronic diseases in elderly people [12, 13]. It was established that low-grade inflammation is linked to atherosclerosis, heart failure, metabolic syndrome, osteoporosis, and dementia and may contribute to the progression of chronic diseases [14, 15]. Physical activity reduces inflammation, and even though the mechanisms are not fully explained, it is known that exercise-induced increased production of anti-inflammatory cytokines protects against chronic inflammation [16, 17].

Stessman et al. [18] demonstrated that even in advanced old age (85-year-old subjects), physical activity is associated with better functioning, health status, and decreased mortality. It was suggested that regular exercise may be an antiageing factor by upregulating various antioxidant systems including antioxidant enzymes [9, 19–21].

Radak et al. [22] observed that reaction to ROS has a bell-shaped curve, indicating that high concentration of ROS is harmful; however, moderate exercise-induced stress might upregulate anti-inflammatory processes by modulation of some transcriptional factors resulting in better resistance to oxidative stress. Oxidative stress-induced changes in redox potential cause, in consequence, the activation of gene expression [23].

It is well documented that in young people, physical activity increases resistance to oxidative stress but the data concerning the effect of exercise in older populations are inconsistent [19–21]. Undoubtedly, a methodology somewhat difficult to standardize and influenced mainly by heterogeneity of the tested group contributes to that. Therefore, the aim of this study was to determine if markers of oxidative stress and enzymatic defense system of the blood are related to physical activity in old and very old men.

## 2. Materials and Methods

The research was conducted in a clinic that specialized in problems of ageing. Main aspects of the research are issues concerning finding a connection between oxidative stress and ageing. Furthermore, studies regarding the reactive forms of oxygen in etiopathogenesis of old-age diseases are performed. There is also an ongoing project dealing with the quality of life and assessment of the functional and cognitive abilities of geriatric patients. Every effort has been made to include only those who had not yet previously had any diseases influencing the diagnosed oxidative stress. None of the included patients suffered from diabetes or rheumatoid diseases. At the moment of collecting the biological material, there was no inflammation. The statistical analysis included only those patients whose anthropometric data within the selected group (Table 1) were consistent with normal distribution.

The participants were divided into four groups according to the age and level of physical activity: the YN group—inactive 65- to 74-year-old males (*n* = 112), the YA group—active males of the same age (*n* = 46), and the ON (*n* = 128) and OA groups—inactive and active 90- to 99-year-old males (*n* = 41). Active males in both age groups reported that they have engaged in physical activity (e.g., long walks, fitness exercises, gymnastic, swimming, Nordic walking, team games, or other recreational exercises) every day in the past 12 months. The physically inactive groups included subjects who did not engage in any moderate leisure time physical activity (LTPA) in the period of the last 12 months. The only active people participating in the study were those who fulfilled the recommendations of the ACSM (American College of Sports Medicine) position stand. These recommendations can be met through 30 minutes of light to moderate intensity exercise at a minimum of 5 times a week.

The active 65- to 74-year-old males who participated in the study were engaged in moderate leisure time physical activity (LTPA) 12.4 ± 0.9 METs × hour/week (or 744 ± 54 METs × minutes/week), whereas the active 90- to 99-year-old males were engaged in moderate leisure time physical activity (LTPA) 10.4 ± 1.1 METs × hour/week (or 624 ± 66 METs × minutes/week). Physical activity of the tested group was assessed in a subjective manner with the use of SDPAR (Seven-Day Physical Activity Recall) questionnaire, which estimates the physical activity in MET units—MET minute/week (1 MET = 3.5 ml O_2_/kg of body weight/min = 1 kcal/kg/hour = 4.184 kJ/kg/hour). The results allow for a classification of those questioned to one of the three categories of physical activity: low (under 600 MET min/week), average (600-1500 MET min/week), or high (over 1500 MET min/week). The questionnaire assesses the total weekly energy expenditure spent on all types of physical activity. It allows for a distinction between the intensity of efforts being either average or intensive [24–26].

All those who smoked tobacco or drank alcohol were excluded from the selection process of participants for our study.

This project was approved by the Bioethics Commission of Nicolaus Copernicus University in Torun (no. KB 340/2015).

Venous blood was collected from the subjects in the morning before breakfast into heparinized and nonheparinized tubes, and both samples were centrifuged for 10 min at 2500 rpm. The obtained erythrocytes were washed three times in cold 0.9% NaCl. After washing, the erythrocytes were diluted at the ratio of 1 : 1 with twice-distilled water and refrigerated at -70°C pending measurements of antioxidant enzyme activities and malondialdehyde (MDA) concentration. Before analysis, the erythrocyte hemolysates were defrosted and centrifuged for 5 min at 2000 rpm. Antioxidant enzyme activities were measured in the erythrocyte hemolysates. The activity of superoxide dismutase (SOD, EC1.15.1.1) was measured according to the method by Misra and Fridovich [27]. The increase of absorbance was measured at *λ* = 480 nm in relation to the reaction of inhibition of adrenaline autooxidation by SOD in pH = 10.2. The unit of SOD activity (U) was the amount of enzyme, which repressed adrenaline autooxidation by 50%, calculated per g of Hb (U/g Hb). The glutathione peroxidase activity (GPx, EC 1.11.1.9) was measured using Paglia and Valentine's method with *tert*-butyl as a substrate and with glutathione reductase transforming the oxidized glutathione into its reduced form [28]. The measure of enzyme activity was the reduction of absorbance at *λ* = 340 nm. The unit of enzyme activity (U) was the amount of enzyme, which caused oxidation of 1 *μ*mol NADPH in 1 min at 25°C. The activity of GPx was expressed in U/g Hb. The catalase activity (CAT, EC 1.11.1.6) was measured according to Beers and Sizer at *λ* = 240 nm at 25°C [29]. The unit of CAT activity (U) was taken to be the amount of the enzyme, which decomposes 1 g of H_2_O_2_ in 1 min at 25°C in pH = 7.0. The activity of CAT was expressed in U/g Hb. The glutathione reductase activity (GR, EC 1.6.4.2) was assayed at 37°C using the method proposed by Flohé and Günzler, which is based on the measurement of decrease of absorbance at *λ* = 340 nm, caused by oxidation of NADPN in the presence of oxidized glutathione [30]. The unit of enzyme activity (U) was the amount of enzyme, which oxidized 1 *μ*mol of NADPH in 1 min. The enzyme activity was expressed as U/g Hb.

Isoprostanes were designated in plasma using the commercial kit ELISA (Assay Designs ADI-901-091, USA).

Carbonyl groups were determined using ELISA kit for the in vitro determination of protein-bound carbonyls in human serum and plasma from Immundiagnostik AG K 7870, Germany.

The MDA concentration was assayed according to Placer et al. [31] in an erythrocyte hemolysate diluted with distilled water at the ratio of 1 : 50. After the reaction of the hemolysate with thiobarbituric acid in a boiled water bath, the specimens were centrifuged at 1200 rpm for 15 min. During the reaction that occurred in the acid environment in the increased temperature, a colour compound is made, the intensity of which was measured spectrophotometrically at 532 nm. The absorbance was measured at *λ* = 532 nm, and MDA concentration was expressed in *μ*mol/g Hb.

The results were presented as a mean ± standard deviation (SD). Intergroup differences and the impact of the main factors of variation were estimated using ANOVA and Tukey's test for different numbers of cases in the groups. All calculations were performed using Statistica 12.0 (StatSoft, USA). The level of significance was set at *p* < 0.05.

## 3. Results

Body mass, height, and BMI were similar in the examined groups of male subjects, aged 65-74 and 90-99 years (Table 1). Although there were no significant differences in body mass and height between these groups, there was a clear tendency towards lower values of these parameters in the oldest old men compared to the younger old participants. BMI in the younger men (65-74 years old) was slightly higher than 25 indicating the preobese status according to the WHO cut-off classification [32].

As shown in Table 2, all of the assayed antioxidant enzyme activities (CAT, SOD, GPx, and GR) and the SOD/GPx ratios were significantly higher in the active younger old males than in the inactive ones (YA vs. YN). In the group of the oldest old active males (OA), only the GPx activity was significantly higher compared to the inactive oldest old ones (ON). There were no significant differences in CAT, SOD, and GR activities and SOD/GPx ratios between the active and inactive oldest old subjects (OA vs. ON).

At the same time, the observed CAT and GPx activities in the younger old inactive men were significantly lower than those in the oldest old inactive subjects. However, in the younger old active men, CAT, SOD, and GR activities and SOD/GPx ratios were significantly higher compared to the oldest old active participants.

The concentrations of isoprostanes, protein carbonyls, and MDA were significantly lower in both groups of active men, independently of age, compared to the respective inactive subjects (Table 3). Additionally, isoprostanes and protein carbonyl concentrations were lower in both the active and inactive younger old males than in the respective groups of oldest old men (YN vs. ON and YA vs. OA). There were no significant differences in MDA concentrations between both the inactive and active younger old and oldest old men (YN vs. ON and YA vs. OA).

## 4. Discussion

The results concerning the age-related changes of antioxidant enzyme activities in the blood are often controversial. In our studies, antioxidant activities were always higher in active men in comparison to inactive men regardless of their age (Table 2). Only catalase activities increased whereas superoxide dismutase activities decreased together with age of active and inactive men. Simioni et al. suggested that physical activity improves antioxidant defenses and lowers lipid peroxidation levels both in adult and in aged individuals. Elderly physically active individuals show antioxidant activity and lipid peroxidation levels similar to young sedentary subjects, emphasizing the importance of regular physical activity to decelerate the ageing-associated impairment process [33, 34]. In our study, in the case of other antioxidants, the activities varied depending on age and/or physical activity and as other researchers show, the results on antioxidant activities are not consistent. For example, Mecocci et al. [35] demonstrated that erythrocyte SOD activity increased with age and was significantly higher in the group of 81- to 99-year-old subjects compared to the younger groups (<60 and 61-80 years of age). On the other hand, Polidori et al. [36] observed that SOD and GPx activities were similar in different age groups (< 60, 61-81, and 81-99 years of age). However, Kłapcińska et al. [37] noted higher CAT and GR but lower SOD activities in a group of centenarians compared with younger subjects. In addition, Özbay and Dülger [38] observed lower erythrocyte SOD and GPx activities in a group of older subjects (57-71 years) compared to younger participants. The effect of the age-related decrease in SOD activity was confirmed by Mariani et al. [39] which is also consistent with our observations, but simultaneously, CAT and GPx activities were similar between participants aged 70-79 years and >90 years. However, examining 249 healthy subjects of different ages (25-70 years), Mendoza-Nunez et al. [40] showed that erythrocyte GPx activity decreased with age, whereas erythrocyte SOD activity was similar in all age groups. On the other hand, compensatory changes were demonstrated in antioxidant enzyme activities, with a decrease in one enzyme activity linked with an increase in another one [41] Meijer et al. [42] demonstrated that physical activity increased antioxidant potential in both women and men aged 50 years and older. Moreover, differences in the activities of individual antioxidants presented in various publications may result from the health status of people, previous illnesses, chronic disease, family stress situations, etc. [34, 43]. So it is important to continuously update and improve our knowledge on the subject.

In the present study, it was shown that the activities of CAT and GPx were higher in the oldest old inactive males compared to the respective group of younger old subjects, but SOD and GR activities were similar. Radak et al. [22] stressed that increased ROS generation plays a role in the induction of antioxidant enzyme activity. Therefore, it cannot be excluded that the examined oldest old inactive male group experienced oxidative stress-induced adaptations. It has to be clearly stated that the adaptive increase in antioxidant enzyme activities was insufficient to prevent the oxidative stress, because in the oldest old group of inactive participants, oxidative stress markers, i.e., isoprostanes and protein carbonyls, were higher than in the respective younger old group, although the concentration of MDA was similar. This contradicts the study by Özbay and Dülger [38] examining women and men aged 9-71 years, which demonstrated that MDA concentration increased with age.

The present study showed that SOD/GPx activity ratio was similar in both groups of oldest old men, but in the group of active younger old subjects, it was higher compared to the inactive younger old men. Kostka et al. [44] suggested that an imbalance in this ratio results in the accumulation of hydrogen peroxide and may be an important factor of cellular ageing.

The obtained data indicates that oxidative stress increased with age, although physical activity resulted in a decrease of plasma isoprostanes and protein carbonyl concentration. In both younger old inactive and oldest old inactive men, physical activity induced higher erythrocyte GPx activity, whereas in the younger old active men, also SOD, CAT, and GR activities were higher than in the inactive subjects. The reason for the lack of exercise-induced increase of antioxidant enzyme activities (SOD, CAT, and GR) in the oldest old group of male subjects may be the volume of exercise stimuli. The level of physical activity in the oldest old participants was lower than that in the younger old ones as a result of far-reaching effects of ageing (sarcopenia, decrease of aerobic capacity, and fitness) [1].

In conclusion, the current study confirmed that oxidative stress is related to age, although CAT and GPx activities were higher in the inactive oldest old men compared to younger old ones. Physical activity caused a decrease in oxidative stress markers independently of the age and resulted in the increase of GPx activity in both the younger old and the oldest old groups, having a beneficial effect even in very old men. This data suggests that regular physical activity is an important factor for preserving the health status even by very old men. Undoubtedly, the different body composition, in which there is more adipose tissue, influences the lower activity of antioxidative enzymes and significantly higher concentration of examined peroxidative markers (isoprostanes, MDA, and CP) in the YN group. People from this group had higher BMI than others. As clearly stated in different works, this state is a beginning of the development of many free-radical-based diseases such as type 2 diabetes or hypertension [45–47]. In the case of our study, these were the exclusion criteria; however, in the long run, it may be possible for the development of those among the participants of the research.

It may be stated that the obtained results confirm the crucial part of free radicals in the ageing process which is also observed through the increased activity of the examined antioxidative enzymes and a higher concentration of peroxidative markers of protein and lipid structures [48]. Simultaneously, the results show that moderate physical activity significantly lowers the oxidative stress, thereby causing a better antioxidative protection of the active people [49].

## Figures and Tables

**Table 1:** General characteristics of the examined groups (mean ± SD).

Parameter	65-74 years		90-99 years	
	YN (*n* = 112)	YA (*n* = 46)	ON (*n* = 128)	OA (*n* = 41)
Body mass (kg)	81.7 ± 7.4	79.1 ± 7.5	72.2 ± 7.5	64.1 ± 5.5
Body height (cm)	177 ± 5.2	174 ± 5.3	170 ± 4.3	158 ± 6.2
BMI	27.2 ± 4.1	25.0 ± 4.4	24.8 ± 3.1	22.3 ± 3.1
Total cholesterol (mg/dl)	178 ± 14.1	185 ± 12.2	168 ± 9.4	165 ± 10.3

^∗^65-74 years: YN: inactive men; YA: active men; 90-99 years: ON: inactive men; OA: active men.

**Table 2:** Antioxidant enzyme activities in the blood in the examined groups (mean ± SD).

Enzyme	65-75 years		90-99 years	
	YN (*n* = 112)	YA (*n* = 46)	ON (*n* = 128)	OA (*n* = 41)
Catalase (CAT) (U/g Hb)	14.7 ± 0.3^A,E^	18.5 ± 0.5^B^	19.3 ± 0.2	19.9 ± 0.2
Superoxide dismutase (SOD) (U/g Hb)	2456 ± 109^A^	2599 ± 115^C^	2335 ± 119	2495 ± 124
Glutathione peroxidase (GPx) (U/g Hb)	8.9 ± 0.5^A,E^	12.9 ± 0.7	9.8 ± 0.3^B^	11.4 ± 0.2
Glutathione reductase (GR) (U/g Hb)	32.7 ± 2.4^A^	58.3 ± 2.1^D^	32.9 ± 2.0	33.8 ± 3.8
Superoxide dismutase/glutathione peroxidase (SOD/GPx) ratio	208.1 ± 87.1^A^	267 ± 75.9^D^	217.9 ± 45.1	232.26 ± 42.5

^A^Significantly lower in the comparison of YN vs. YA, *p* < 0.01. ^B^Significantly lower in the comparison of YN vs. ON, *p* < 0.05. ^C^Significantly higher in the comparison of OA vs. YA, *p* < 0.05. ^D^Significantly higher in the comparison of OA vs YA, *p* < 0.001. ^E^Significantly lower in the comparison of YN vs. ON, *p* < 0.01.

**Table 3:** Markers of oxidative stress in the examined groups (mean ± SD).

Parameter	65-74 years		90-99 years	
	YN (*n* = 112)	YA (*n* = 46)	ON (*n* = 128)	OA (*n* = 41)
Isoprostanes (pg/ml)	555 ± 42^C^	421 ± 31^A,D^	811 ± 11	677 ± 9^B^
Malondialdehyde (MDA) (*μ*mol/g Hb)	0.25 ± 0.02	0.23 ± 0.03^A^	0.25 ± 0.03	0.23 ± 0.01^B^
Protein carbonyls (CP) (pmol/mg protein)	299 ± 19^C^	206 ± 11^A,D^	608 ± 20	516 ± 9^B^

^A^Significantly lower in the comparison of YA vs. YN, *p* < 0.01. ^B^Significantly lower in the comparison of ON vs. OA, *p* < 0.05. ^C^Significantly lower in the comparison of YN vs. ON, *p* < 0.001. ^D^Significantly lower in the comparison of YA vs. OA, *p* < 0.001.

## Data Availability

The datasets generated and/or analysed during the current study can be obtained and is available from the corresponding author on request from other scientists.

## References

[1] Batt M. E., Tanji J., Börjesson M. (2013). Exercise at 65 and beyond. *Sports Medicine*.

[2] Zapata-Diomedi B., Gunn L., Giles-Corti B., Shiell A., Lennert Veerman J. (2018). A method for the inclusion of physical activity-related health benefits in cost-benefit analysis of built environment initiatives. *Preventive Medicine*.

[3] Borjesson M., Urhausen A., Kouidi E. (2011). Cardiovascular evaluation of middle-aged/senior individuals engaged in leisure-time sport activities: position stand from the sections of exercise physiology and sports cardiology of the European Association of Cardiovascular Prevention and Rehabilitation. *European Journal of Cardiovascular Prevention & Rehabilitation*.

[4] Ebell M. H. (2017). Combination of resistance and aerobic exercise best for older persons with obese. *American Family Physician*.

[5] Monteiro-Junior R. S., de Tarso Maciel-Pinheiro P., da Matta Mello Portugal E. (2018). Effect of exercise on inflammatory profile of older persons: systematic review and meta-analyses. *Journal of Physical Activity & Health*.

[6] Silva R. B., Aldoradin-Cabeza H., Eslick G. D., Phu S., Duque G. (2017). The effect of physical exercise on frail older persons: a systematic review. *The Journal of Frailty & Aging*.

[7] Kregel K. C., Zhang H. J. (2007). An integrated view of oxidative stress in aging: basic mechanisms, functional effects, and pathological considerations. *American Journal of Physiology-Regulatory, Integrative and Comparative Physiology*.

[8] Pomatto L. C. D., Davies K. J. A. (2018). Adaptive homeostasis and the free radical theory of ageing. *Free Radical Biology & Medicine*.

[9] Goto S., Naito H., Kaneko T., Chung H. Y., Radák Z. (2007). Hormetic effects of regular exercise in aging: correlation with oxidative stress. *Applied Physiology, Nutrition, and Metabolism*.

[10] Yamada T., Steinz M. M., Kenne E., Lanner J. T. (2017). Muscle weakness in rheumatoid arthritis: the role of Ca^2+^ and free radical signaling. *EBioMedicine*.

[11] Hensley K., Floyd R. A. (2002). Reactive oxygen species and protein oxidation in aging: a look back, a look ahead. *Archives of Biochemistry and Biophysics*.

[12] Woods J. A., Wilund K. R., Martin S. A., Kistler B. M. (2012). Exercise, inflammation and aging. *Aging and Disease*.

[13] Baierle M., Nascimento S. N., Moro A. M. (2015). Relationship between inflammation and oxidative stress and cognitive decline in the institutionalized elderly. *Oxidative Medicine and Cellular Longevity*.

[14] Lee R., Margaritis M., Channon K. M., Antoniades C. (2012). Evaluating oxidative stress in human cardiovascular disease: methodological aspects and considerations. *Current Medicinal Chemistry*.

[15] Nerpin E., Helmersson-Karlqvist J., Risérus U. (2012). Inflammation, oxidative stress, glomerular filtration rate, and albuminuria in elderly men: a cross-sectional study. *BMC Research Notes*.

[16] Beavers K. M., Brinkley T. E., Nicklas B. J. (2010). Effect of exercise training on chronic inflammation. *Clinica Chimica Acta*.

[17] Chen L., Deng H., Cui H. (2018). Inflammatory responses and inflammation-associated diseases in organs. *Oncotarget*.

[18] Stessman J., Hammerman-Rozenberg R., Cohen A., Ein-Mor E., Jacobs J. M. (2009). Physical activity, function, and longevity among the very old. *Archives of Internal Medicine*.

[19] Radak Z., Torma F., Berkes I. (2019). Exercise effects on physiological function during aging. *Free Radical Biology and Medicine*.

[20] Sellami M., Gasmi M., Denham J. (2018). Effects of acute and chronic exercise on immunological parameters in the elderly aged: can physical activity counteract the effects of aging?. *Frontiers in Immunology*.

[21] Coelho-Júnior H. J., Rodrigues B. (2017). Exercise and aging: different approaches to different beneficial effects. *Gerontology and Geriatric Medicine*.

[22] Radak Z., Chung H. Y., Goto S. (2005). Exercise and hormesis: oxidative stress-related adaptation for successful aging. *Biogerontology*.

[23] Yu B. P., Chung H. Y. (2006). Adaptive mechanisms to oxidative stress during aging. *Mechanisms of Ageing and Development*.

[24] Calfas K. J., Sallis J. F., Nichols J. F. (2000). Project GRAD: two-year outcomes of a randomized controlled physical activity intervention among young adults. Graduate ready for activity daily. *American Journal of Preventive Medicine*.

[25] Sallis J. F., Calfas K. J., Nichols J. F. (1999). Evaluation of a university course to promote physical activity: project GRAD. *Research Quarterly for Exercise and Sport*.

[26] Hayden-Wade H. A., Coleman K. J., Sallis J. F., Armstrong C. (2003). Validation of the telephone and in-person interview versions of the 7-day PAR. *Medicine and Science in Sports and Exercise*.

[27] Misra H. P., Fridovich I. (1972). The role of superoxide anion in the autoxidation of epinephrine and a simple assay for superoxide dismutase. *The Journal of Biological Chemistry*.

[28] Paglia D. E., Valentine W. N. (1967). Studies on the quantitative and qualitative characterization of erythrocyte glutathione peroxidase. *The Journal of Laboratory and Clinical Medicine*.

[29] Beers R. F., Sizer I. W. (1952). A spectrophotometric method for measuring the breakdown of hydrogen peroxide by catalase. *The Journal of Biological Chemistry*.

[30] Flohé L., Günzler W. A. (1984). [12] Assays of glutathione peroxidase. *Methods in Enzymology*.

[31] Placer Z. A., Cushman L. L., Johnson B. C. (1966). Estimation of product of lipid peroxidation (malonyl dialdehyde) in biochemical systems. *Analytical Biochemistry*.

[32] WHO (2011). *Obesity and Overweight*.

[33] Rowiński R., Kozakiewicz M., Kędziora-Kornatowska K., Hübner-Woźniak E., Kędziora J. (2013). Markers of oxidative stress and erythrocyte antioxidant enzyme activity in older men and women with differing physical activity. *Experimental Gerontology*.

[34] Simioni C., Zauli G., Martelli A. M. (2018). Oxidative stress: role of physical exercise and antioxidant nutraceuticals in adulthood and aging. *Oncotarget*.

[35] Mecocci P., Polidori M. C., Troiano L. (2000). Plasma antioxidants and longevity: a study on healthy centenarians. *Free Radical Biology and Medicine*.

[36] Polidori M. C., Mariani E., Baggio G. (2007). Different antioxidant profiles in Italian centenarians: the Sardinian peculiarity. *European Journal of Clinical Nutrition*.

[37] Kłapcińska B., Derejczyk J., Wieczorowska-Tobis K., Sobczak A., Sadowska-Krepa E., Danch A. (2000). Antioxidant defense in centenarians (a preliminary study). *Acta Biochimica Polonica*.

[38] Özbay B., Dülger H. (2002). Lipid peroxidation and antioxidant enzymes in Turkish population: relation to age, gender, exercise, and smoking. *The Tohoku Journal of Experimental Medicine*.

[39] Mariani E., Cornacchiola V., Polidori M. C. (2006). Antioxidant enzyme activities in healthy old subjects: influence of age, gender and zinc status. *Biogerontology*.

[40] Mendoza-Núñez V. M., Ruiz-Ramos M., Sánchez-Rodríguez M. A., Retana-Ugalde R., Muñoz-Sánchez J. L. (2007). Aging-related oxidative stress in healthy humans. *The Tohoku Journal of Experimental Medicine*.

[41] Hübner-Woźniak E., Okecka-Szymańska J., Stupnicki R., Malara M., Kozdroń E. (2011). Age-related blood antioxidant capacity in men and women. *Journal of Medical Biochemistry*.

[42] Meijer E. P., Goris A. H. C., van Dongen J. L. J., Bast A., Westerterp K. R. (2002). Exercise induced oxidative stress in older adults as a function of habitual activity level. *Journal of the American Geriatrics Society*.

[43] Dudzińska E., Gryzinska M., Ognik K., Gil-Kulik P., Kocki J. (2018). Oxidative stress and effect of treatment on the oxidation product decomposition processes in IBD. *Oxidative Medicine and Cellular Longevity*.

[44] Kostka T., Drai J., Berthouze S., Lacour J. R., Bonnefoy M. (1998). Physical activity, fitness and integrated antioxidant system in healthy active elderly women. *International Journal of Sports Medicine*.

[45] Di Segni C., Silvestrini A., Fato R. (2017). Plasmatic and intracellular markers of oxidative stress in normal weight and obese patients with polycystic ovary syndrome. *Experimental and Clinical Endocrinology & Diabetes*.

[46] Kim M., Paik J. K., Kang R., Kim S. Y., Lee S. H., Lee J. H. (2013). Increased oxidative stress in normal-weight postmenopausal women with metabolic syndrome compared with metabolically healthy overweight/obese individuals. *Metabolism*.

[47] Di Renzo L., Galvano F., Orlandi C. (2010). Oxidative stress in normal-weight obese syndrome. *Obesity*.

[48] Peluso I., Palmery M., Yarla N. S., Perry G., Kamal M. A. (2018). From oxidative stress to ageing via lifestyle, nutraceuticals, polypharmacy, and neuropsychological factors. *Oxidative Medicine and Cellular Longevity*.

[49] Ihle A., Gouveia É. R., Gouveia B. R. (2017). The role of leisure activities in mediating the relationship between physical health and well-being: differential patterns in old and very old age. *Gerontology*.

